# Intersectoral interventions for people living with obesity: a scoping review and bibliometric analysis

**DOI:** 10.1186/s12889-026-27953-6

**Published:** 2026-05-30

**Authors:** Géraldine Layani, Sopie Marielle Yapi, Anne Schweitzer, Thameya Balasingam, Laurence Berthelet, Frédéric Bergeron, Jean-Baptiste Gartner, Audrey L’Espérance, Maxime Sasseville, Lily Lessard, André Coté, Antoine Boivin, Brigitte Vachon, Blandine Lentilhac, Nadia Sourial

**Affiliations:** 1 Centre de Recherche du Centre Hospitalier de l’Université de Montréal, Université de Montréal, Montréal, QC Canada; 2 Department of Family and Emergency Medicine, Faculty of Medicine, Université de Montréal, P.O. Box 440 Laval, 1755 René-Laennec Boulevard, Montréal, Québec, QC H7M 3L9 Canada; 3 Université du Québec en Outaouais, Gatineau, QC Canada; 4 Centre de recherche du CISSS de Chaudière-Appalaches, Chaudière-Appalaches, Lévis, QC Canada; 5 Bibliothèque, Direction des services-conseils, Université Laval, Québec, QC Canada; 6 Centre de recherche en gestion des services de santé, Université Laval, Québec, QC Canada; 7 Département de management, Université Laval, Québec, QC Canada; 8 VITAM Centre de Recherche en Santé durable, Québec, QC Canada; 9 Faculté des sciences infirmières, Université Laval, Québec, QC Canada; 10 École Nationale d’Administration Publique, Québec, QC Canada; 11 Interdisciplinary Chair in Health and Social Services for Rural Populations, Université du Québec à Rimouski, Rimouski, QC Canada; 12 Faculty of Medicine, Université de Montréal, Montréal, QC Canada; 13 Department of Health Management, Evaluation and Policy, School of Public Health, Université de Montréal, Montréal, QC Canada

**Keywords:** Obesity, Intersectoral collaboration, Community health workers, Determinants of health, Chronic disease, Community-based care

## Abstract

**Background:**

Obesity is increasingly recognized as a complex chronic condition shaped by genetic, behavioural, social, and environmental factors. While intersectoral collaboration is considered essential to address these multifaceted drivers, few studies have systematically explored the nature and implementation of such interventions, particularly outside the clinical domain.

**Objective:**

This study aimed to identify and characterize intersectoral health interventions targeting people living with obesity within community and primary care settings, drawing from both scientific and grey literature.

**Methods:**

Following Arksey and O’Malley’s framework, we conducted a scoping review searching across three academic databases (MEDLINE, Web of Science, and CINAHL) and multiple grey literature sources. Inclusion criteria focused on intersectoral interventions developed from 2006 to 2025, targeting adults living with obesity in high-income countries. Data extraction was guided by a concept map of determinants of health (DoH) developed in collaboration with patient-partners. A bibliometric analysis of included studies was also conducted using the VOSviewer^®^ software.

**Results:**

Among 23 included records (20 peer-reviewed and 3 grey literature), most interventions originated from the United States and focused on lifestyle modification. While none explicitly defined “intersectorality,” collaborations typically involved healthcare, academic, community, and faith-based organizations. Community health workers emerged as central actors linking clinical and social domains. Interventions often targeted minority populations and emphasized cultural relevance. However, outcome measures remained largely biomedical, with limited integration of DoH indicators.

**Conclusion:**

Intersectoral interventions for people living with obesity remain under-conceptualized, despite growing implementation. To foster equity and person-centered care, future efforts must embed DoH into evaluation frameworks, support community health workers as structural agents of integration, and formalize intersectoral governance. Co-designed approaches grounded in community realities offer promising pathways for sustainable and inclusive obesity management strategies.

**Supplementary Information:**

The online version contains supplementary material available at 10.1186/s12889-026-27953-6.

## Introduction

Obesity has emerged as a global public health crisis, characterized by its complex and multifactorial etiology [1]. Obesity extends beyond a mere measure of body weight and represents a complex condition shaped by the dynamic interplay of multiple determinants of health (DoH) across the life trajectory, including genetic predispositions, social and commercial determinants of health (SDoH, CDoH), environmental contexts, and individual health behaviors [2–8]. Despite its formal recognition as a chronic disease by the Canadian Medical Association in 2006, societal perceptions continue to oscillate between medical and social framings [9, 10]. Recent expert panels have sought to clarify this complexity by distinguishing between preclinical obesity (as a risk factor) and clinical obesity (as a disease entity), thereby better guiding both clinical and policy responses [11]. Current interventions remain predominantly weight-centric, with limited responsiveness to the lived experiences of people living with obesity (PLO) and the broader DoH shaping their health trajectories [10, 12–15].

Given that the health sector acts only modestly (approximately 20%) on the determinants affecting the health of PLO [15–17], effective management of obesity requires a shift toward an intersectoral approach that takes into account the patient’s entire life trajectory and integrates various levers of action across different sectors/domains [18, 19]. Intersectoral collaboration, as defined by the World Health Organization, entails the involvement of multiple sectors in designing and implementing public policies to enhance health, equity, and well-being [18, 20]. This collaboration can take diverse forms, ranging from networking to full co-construction [21]. However, most existing interventions remain confined within the healthcare sector, underutilizing the transformative potential of intersectoral action [10]. Although this principle is not new, as illustrated by frameworks such as Health in All Policies and Healthy Cities, its operationalization in the context of obesity remains limited and insufficiently documented [22–25]. 

In this context, the citizen-based participatory action research project COLLAB-INTER-360-Obesity was launched in 2023 with the aim of co-developing, together with PLO and stakeholders from diverse sectors of society, an intersectoral learning community [26]. This community brings together participants from the health, community, political, civil society, and academic sectors to enhance the support provided to PLO within their communities and across their life trajectories. Given the limited understanding of how participants from different sectors contribute to such a learning community, and the lack of a well-defined body of literature on the role of intersectoral collaboration in health interventions, a scoping review was deemed necessary as a preliminary step [27]. This review was intended to provide a more straightforward overview of the components and characteristics of interventions that are both useful and structured for building the learning community. Accordingly, the present study aimed to identify and characterize intersectoral interventions targeting PLO in community settings and primary care, using a hybrid method combining a scoping review of peer-reviewed and grey literature and a bibliometric analysis of terms included in peer-reviewed literature.

## Methods

### A- scoping review

The study followed a previously published protocol on BMJ Open [28]. The scoping review was developed based on Arksey and O’Malley’s methodological framework using four steps: (1) search strategies and data sources, (2) selection process, (3) data extraction and (4) data synthesis [29]. The research question was: “What intersectoral health interventions have been developed within the community and primary care settings for people living with obesity?” Based on this question, the following secondary question will be examined: “What are the characteristics of these interventions?”

1. Search Strategies and Data Sources

The scoping review was conducted between May 2024 and February 2025. Initially, an exploratory search was conducted using MEDLINE to identify relevant search terms from text words, subject headings (including MeSH terms), and author-assigned keywords in relevant publications—the final search combined two core concepts: intersectorality and obesity. The search strategy was then tailored for each database. The published literature was searched using the following electronic databases: MEDLINE (PubMed), Web of Science, and Cumulative Index to Nursing and Allied Health Literature (CINAHL). These databases offer comprehensive indexing of multidisciplinary health literature and represent the standard for evidence synthesis in public health [30]. Grey literature was also consulted using the metasearch engine “eTools.ch” to identify relevant documents and websites. We also launched sub-searches in various regionalized Google searches (google.fr, google.ca, etc.), limiting the number of results to a maximum of 50 for each. Additionally, catalogues (Santécom, BANQ), and a list of preidentified organizations (Obesity Canada, etc.) were consulted to identify reports relevant to this scan. The full search strategy for each database and resource is provided in Supplemental Material 1 and the protocol [28].

2. Selection Process

For the peer-reviewed literature, all identified records were imported into Covidence for screening and data extraction [31]. The software automatically removed duplicate records, with any remaining mismatches resolved through manual review. Eligibility assessment was conducted independently by four reviewers from the research team (MY, TB, AS, LB) using a two-stage screening process. First, reviewers evaluated records based on titles and abstracts, followed by full-text assessment.

Regarding the grey literature, we compiled a bilingual Excel file (French and English) of the sites consulted. Duplicates were eliminated using Excel’s built-in functions. A first reviewer examined the sites written in French, while a second evaluated those in English. Subsequently, the roles were reversed to ensure a second cross-reading of all the sites.

Throughout the screening process, reviewers met regularly to discuss discrepancies and maintain consistency in the application of the inclusion criteria. Any unresolved disagreements were settled through discussion with the lead researcher (GL).

#### Eligibility criteria

Studies were included if they: (1) described intersectoral interventions for PLO in community/primary care settings. We considered an intervention to be intersectoral when it involved an interaction between the primary healthcare sector and one or more sectors of society to achieve better health outcomes in PLO; (2) targeted adults (≥ 18 years) living with obesity; (3) were published between 2006 and 2024 (post-recognition of obesity as a chronic disease); (4) were published in English or French; and (5) originated from Australia, Canada, France, Germany, the Netherlands, New Zealand, Norway, Sweden, Switzerland, the United Kingdom, and the United States. The selection of the 11 high-income countries was informed by the Commonwealth Fund’s International Health Policy framework, which provides a standardized basis for comparing health system performance across nations with comparable health expenditures and infrastructures [32].

3. Data extraction

Two reviewers independently extracted data from eligible studies using a standardized form developed by the research team, and any discrepancies were resolved through discussion. The information to be extracted encompasses source details and intervention characteristics, including the level of action, target population, the involved sectors and actors, and DoH, as outlined in a concept map developed by the research team. Details of the information collected can be found in the protocol [28]. 

#### Concept map of Determinants of Health (DoH)

The Wisconsin Framework, initially chosen in the protocol to extract DoH [17], proved insufficient to comprehensively identify and categorize health and health care, socioeconomic, political, cultural, commercial and environmental factors that influence population health, particularly for people living with chronic conditions such as obesity. Indeed, these determinants frequently interact within complex and dynamic systems [33]. Thus, a more nuanced framework that better captures the individual and community-level dimensions of health determinants was developed by the research team, enabling efficient extraction and synthesis of results.

**Fig. 1:**
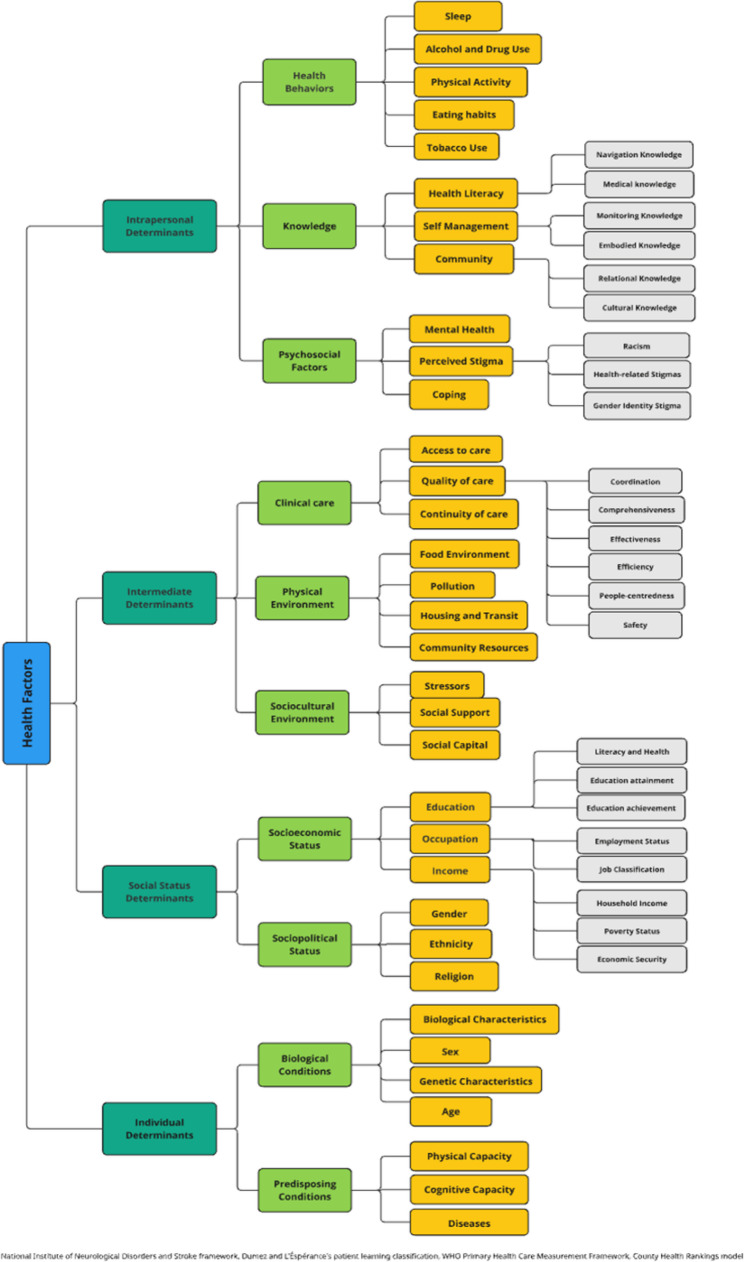
Concept map of determinants of health

Following several in-depth discussions, the research team conducted an evidence synthesis in collaboration with a research librarian (RO) to identify additional suitable frameworks and further refine DoH classification. This process involved a dual-track approach: first, an analysis of 11 articles utilizing the Wisconsin County Health Rankings as a reference; and second, a broader search using Medline that identified 24 conceptual frameworks addressing the factors influencing global health (See supplemental material 6 for search strategy). From this pool of articles, three frameworks were selected for their thematic relevance in consultation with the team: the National Institute of Neurological Disorders and Stroke framework [34], Dumez and L’Éspérance’s patient learning classification [35], and the WHO Primary Health Care Measurement Framework [36]. The synthesis was finalized through a deliberative workshop where three patient-partners (ML, MT, LB) and four members of the research team (GL, AS, MY, TB) reviewed the retained frameworks to strengthen the County Health Rankings model within the Wisconsin framework [37]. Fig. 1 presents the developed concept map.

4. Data synthesis

Results were reported using a PRISMA-ScR (Preferred Reporting Items for Systematic reviews and Meta-Analyses – extension for Scoping Reviews) 2020 flow diagram, which documented the screening stages from identification to final inclusion and also mobilized the PRISMA 2020 checklist (Supplemental Material 2) [38]. The characteristics of the included interventions were summarized in tables.

### B- Bibliometric analysis

**Fig. 2:**
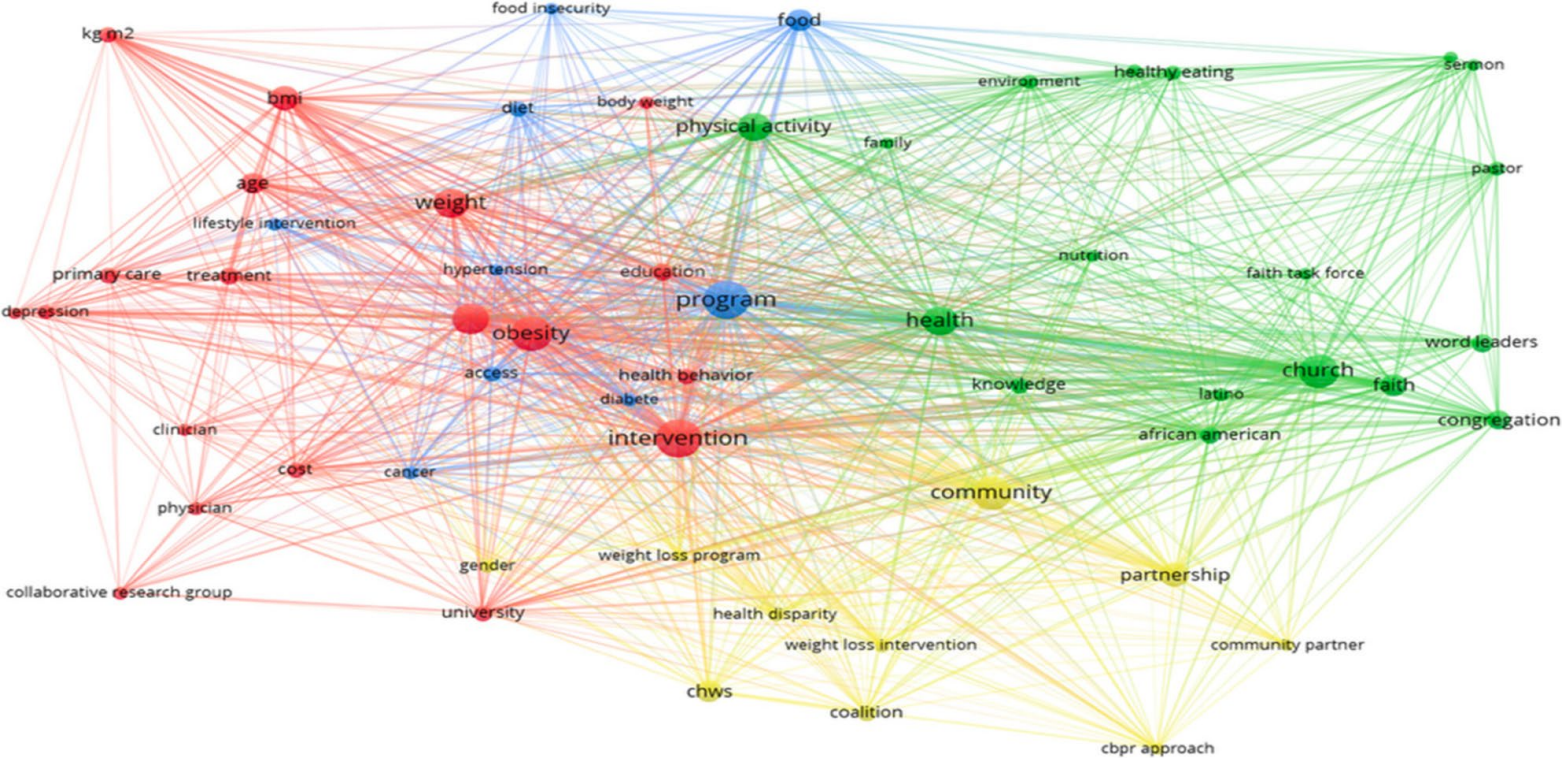
Bibliometric map

As the scoping review methodology presents some limitations on the qualitative analysis of literature [39], the research team conducted a bibliometric network analysis by integrating all 20 peer-reviewed articles, excluding abstracts and bibliographies, into the VOSviewer software (version 2025) [40]. The 20 full texts resulted in 7,929 terms, from which 55 with a minimum occurrence of 15 were selected. The mapping process is automated and algorithmic, ensuring that the spatial distribution, clustering, and node placement are determined by the software’s co-occurrence frequency and not by manual researcher intervention. The program uses association strength to group terms that have a higher density of links (frequency with which they appear together) between them compared to the rest of the network. The results helped us refine our findings from the scoping review by defining the links between keywords and their occurrences [41]. Fig. 2 shows the full bibliometric map and Supplemental Material 3 presents the list of terms included in the map.

The bibliometric map represents the frequency of terms appearing in the 20 peer-reviewed articles selected for the scoping review and how they are connected. The size of the word and node is proportional to the frequency of the term; the distance between nodes and the thickness of the lines represent the link strength between two terms (co-occurrence frequency). It means that closer nodes with thicker lines indicate terms that appear together more frequently in the literature. The central concepts “obesity”, “health”, “intervention”, and “program” are the largest, surrounded by related terms concerning interventions, participants, and stakeholders. The different color clusters suggest four distinct conceptual groupings automatically identified by the VOSviewer clustering algorithm.

## Results

**Fig. 3:**
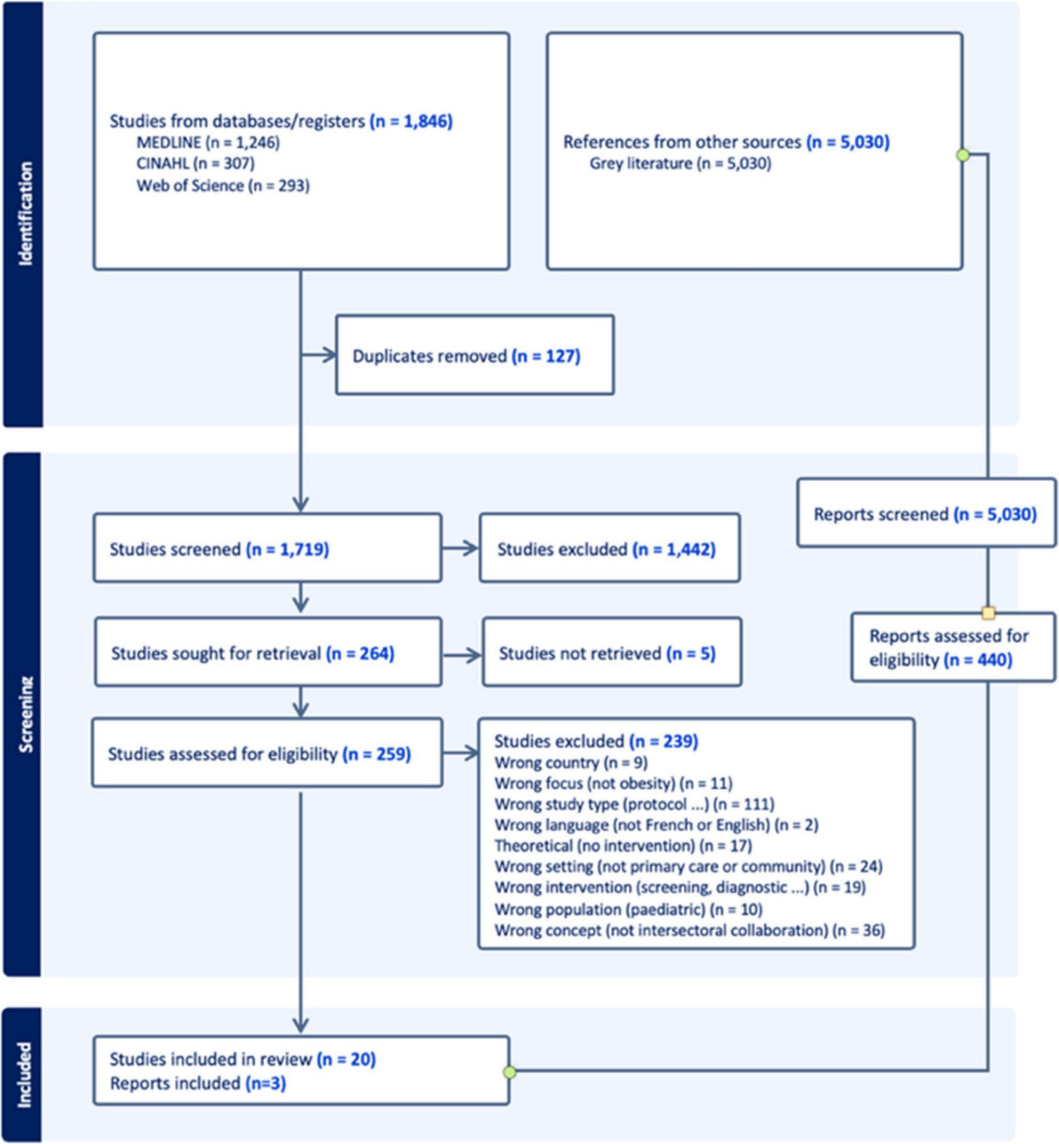
PRISMA-ScR flow diagram

The search retrieved 1,246 articles from Medline, 307 articles from CINAHL, and 293 articles from the Web of Science, for a total of 1,719 after duplicates were removed. Following screening by title and abstract, 259 studies were included in the full-text review from which 20 were selected. The main reason for exclusion at the full-text stage was that article used an ineligible study design (e.g. conference abstracts, editorials). Regarding the grey literature, we consulted a total of 5,030 sites and selected 440. Following the title and document review, three documents met the inclusion criteria. Ultimately, we identified 23 records that met all eligibility criteria. The screening process is further detailed in the PRISMA-ScR flow diagram (Fig. 3).

### Study characteristics

A summary of the 23 records included in this review is presented in Table 1. Selected records were published between 2008 and 2025, with most studies conducted in the United States (*n* = 19). Among the 20 peer-reviewed articles included in the review, 12 were interventional studies (6 randomized controlled trials, 2 community-based participatory research studies, 1 non-randomized experimental study, 1 clinical trial, and 2 pre-post studies), and 8 were observational studies (4 case studies and 4 descriptive studies). Eleven studies were conducted in rural settings, seven in urban settings, and one at the state level. (See Supplemental Material 4)

**Table 1:** Characteristics of included records

Lead author	Year	Country	Design	Aim	Definition of obesity
Peer-reviewed literature					
Bradley [42]	2009	United States	Descriptive study	To describe the effects of a lifestyle modification program on short-term weight loss in a morbidly obese population.	BMI ≥ 30 kg/m2
Brink [43]	2022	Netherlands	Descriptive study	To use a system view on overweight, complexity science, and a transdisciplinary process to develop a five-year integrative obesity-coaching and research program.	BMI ≥ 30 kg/m2 and 4 Chinese subtypes
Derose [44]	2018	United States	Community based participatory research	To describe our experience developing a multi-ethnic, multi-denominational faith and public health partnership to address health disparities.	BMI ≥ 30 kg/m2
Dorling [45]	2022	United States	Randomized controlled trial	To identify the mediators of weight change during an ILI compared with usual care (UC) in underserved patients with obesity.	BMI ≥ 30–50 kg/m2
Elder [46]	2013	United States	Descriptive study	To identify the best approach for promoting health in their own communities.	N/A
Ely [47]	2008	United States	Randomized controlled trial	To evaluate the feasibility of the CCM program and to assess effect sizes for future definitive trials of the intervention.	BMI ≥ 30 kg/m2
Goldfinger [48]	2008	United States	Pre-post study	To test the effectiveness of a peer-led weight loss course	BMI ≥ 30 kg/m2
Horton [49]	2013	United States	Descriptive study	To describe the partnership between a predominantly African American church with a state university nursing school to reduce obesity through a health-promotion program.	N/A
Kim [50]	2008	United States	Community based participatory research	To describe a faith-based weight loss intervention using a community-based participatory research approach in a rural African American faith community	N/A
Leng [51]	2020	United States	Case control study	To discuss strengths and challenges posed by a multi-sector partnership	N/A
Murrock [52]	2010	United States	Randomized controlled trial	To evaluate a culturally specific dance intervention	BMI ≥ 30 kg/m2, % body fat ≥ 35% (F) and 25% (M)
Oliveira [53]	2021	United States	Case report	To describe a pilot food prescription program	BMI ≥ 30 kg/m2
Payan [54]	2021	United States	Randomized controlled trial	To describe the development and implementation of healthy eating and physical activity sermons	N/A
Simonsen [55]	2017	United States	Randomized controlled trial	To address obesity-related health disparities impacting Utah women of color	N/A
Venditti [56]	2021	United States	Clinical trial	To highlight the empirical findings of eight RAINBOW research papers and discuss implications	BMI ≥ 35 kg/m2
Wilson [57]	2010	United States	Non-randomized experimental study	To test a clinician-delivered intervention that utilized community resources for in-depth counseling for unhealthy behaviors including overweight	N/A
Wise-Thomas [58]	2023	United States	Pilot study	To test the feasibility of evaluating HELP-PC in a rural community	BMI
Yeary [59]	2011	United States	Pre-post intervention	To determine if innovative approaches that are sensitive to rural African Americans to address obesity are efficient	BMI
Yeary [60]	2014	United States	Randomized controlled trial	To describe the study development, purpose, and methods of the WORD study.	BMI
Yeh [61]	2010	United States	Case study	To describe the common and distinct features of the three trials; key processes and characteristics of this Collaborative Research Group; and discuss the advantages and disadvantages of this novel organizational approach to organizing multi-center efforts	BMI ≥ 30–50 kg/m2
Grey literature					
Association pour la santé publique du Québec et Coalition québécoise sur la problématique du poids [62]	2021	Canada	Report	To describe the process to create a guide	BMI/Risk factor/Disease
Health Consumers’ Council [63]	2025	Australia	Website	To give information on how you can get involved in the council	N/A
NHS England [64]	2022	UK	Website	To give access and information about the program	BMI ≥ 30 kg/m2

### Obesity

**Fig. 4:**
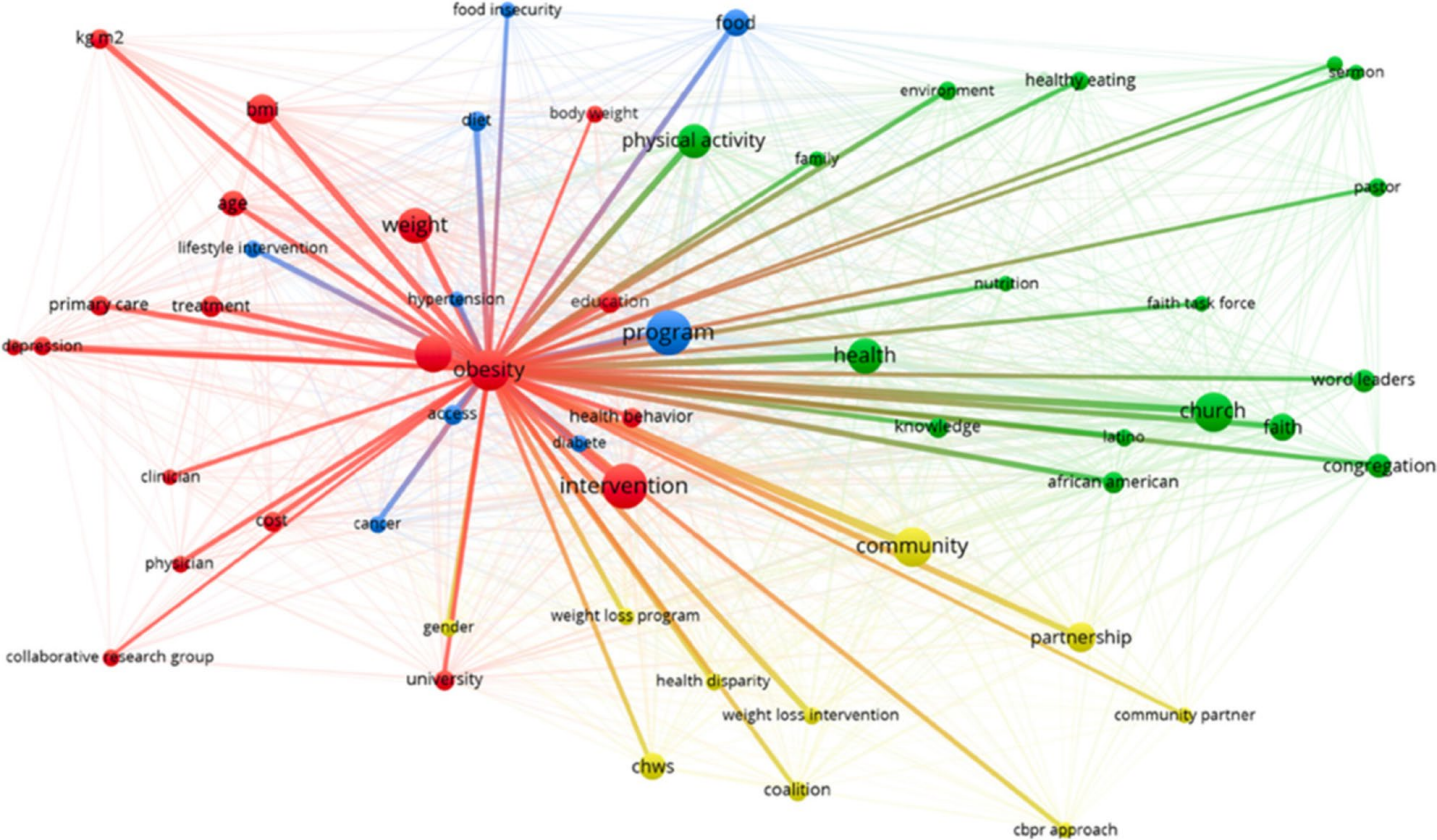
Obesity bibliometric links

The bibliometric analysis shown in Fig. 4 corroborated the data extraction presented in Table 1. Indeed, the prominence of the “weight” node (*n* = 196) and its close proximity to “obesity” reflects a strong thematic focus on anthropometric metrics such as “BMI” (*n* = 116) and “kg/m^2^” (*n* = 38). As detailed in Table 1, the majority of studies used a BMI ≥ 30, and two studies also used a BMI of 50 as an upper limit for participant inclusion. One of the studies, in addition to using the BMI, employed the four Chinese subtypes for obesity to subclassify study participants further and individualize their care. Since BMI can lead to misclassification by not directly measuring body fat, one study also used the percentage of body fat, in addition to BMI, to diagnose obesity. Other studies required the presence of a weight-related cardiovascular risk factor, such as hypertension, dyslipidemia, or diabetes, for study participation. Figure 4 indicate that the main terms linked to obesity are rooted in the biomedical model. The map highlights a high density of co-occurrence between “obesity” and a broad network of clinical terms within the Red Cluster (“clinician,” “physician,” “primary care,” “usual care,” “treatment”) and health conditions (“hypertension,” “depression,” “cancer,” “diabetes”). While social, commercial and environmental factors appear in a relative peripheral positioning within the Green and Blue Clusters, showing a lower frequency of co-occurrence with “obesity”.(See Supplemental Material 3 for full list of terms).

Regarding the grey literature, one source defined obesity in terms of BMI. One did not define obesity, and the last one did not take a position. The authors argue that the accuracy and relevance of BMI are questionable, as it does not measure body fat. They also note that while some experts advocate for recognizing obesity as a chronic disease, others view it primarily as a risk factor.

## Determinants of health

**Fig. 5:**
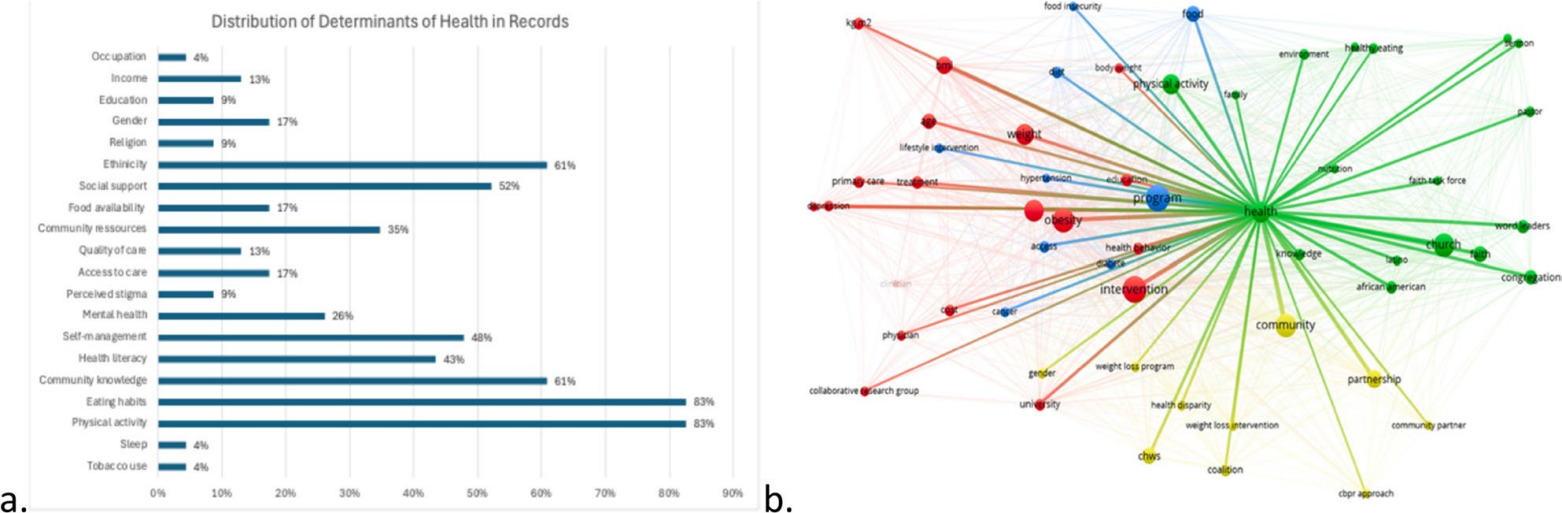
Distribution of determinants of health (DoH) and Health bibliometric links

DoH play a crucial role in the development of obesity, extending beyond the realm of medical care. As shown by the frequency of DoH extracted in records represented in Fig. 5.a, interventions selected in the scoping review were primarily focused on physical activity and eating habits (including food and nutrition), followed by ethnicity (African American and Latino), community knowledge, social support, self-management, health literacy, and community resources. Few interventions addressed socioeconomic determinants (e.g., occupation, income). Ethnicity was prevalent, as many interventions were addressed to minorities, suggesting that interventions are responsive to the needs of the most affected populations. In Fig. 5.b, the enlarged node for “health” serves as a central hub. Many DoH-related terms—such as “physical activity,” “knowledge,” “nutrition,” “food insecurity,” “social support,” “family,” “community,” and “environment”—are prominent and have strong connections to the “health” node forming the Green Cluster. The juxtaposition of the distribution of DoH extracted from the scoping review and the health bibliometric links shows strong alignment with terms from the text represented in the bibliometric map.

### Intersectoral collaboration

Despite the central focus on intersectorality, no study explicitly defined the term. Studies employed other names/concepts closely related to intersectoral collaboration, to develop and implement interventions. The most frequently used terms identified in the bibliometric map (see Fig. 2) were community-based participatory research (CBPR) approach (*n* = 19), partnerships (*n* = 115), and coalitions (*n* = 41).

**Fig. 6:**
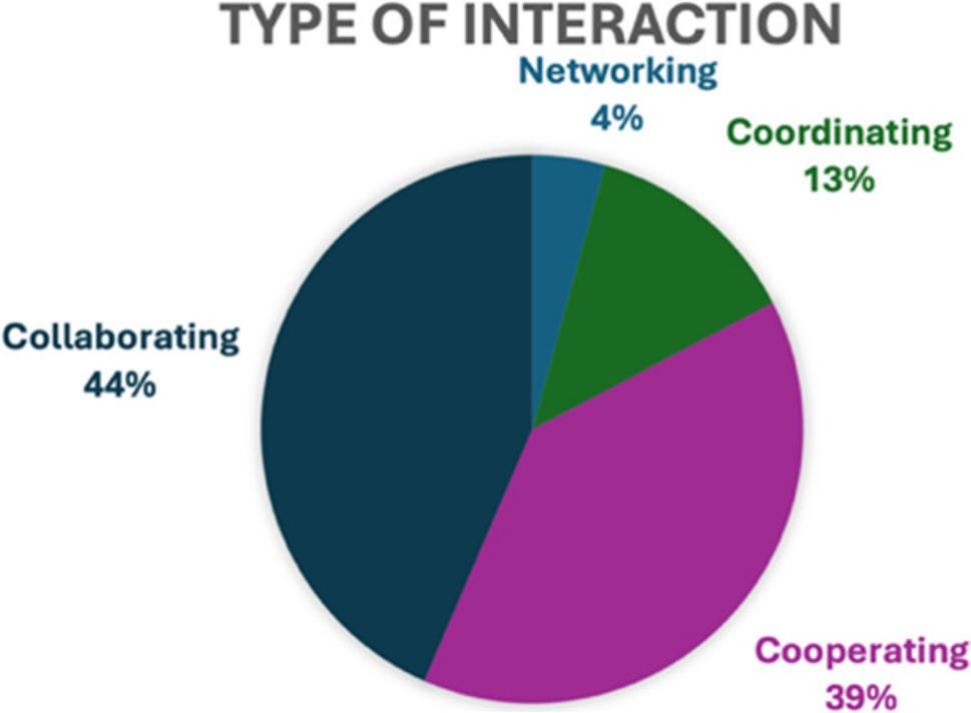
Distribution of types of interaction

Figure 6 illustrates the distribution of interaction types employed in selected interventions. Most often, actors from different sectors cooperated (*n* = 9) or collaborated (*n* = 10) to offer interventions tailored to the needs of PLO. The latter are the most integrated forms of intersectoral collaboration, which involve a close partnership between sectors, where participating organizations work together and share their resources.

### Characteristics of interventions

Supplemental material 4 presents the full table of extracted data. All interventions were aimed at achieving weight loss, primarily through weight loss programs and lifestyle modification programs that increased levels of physical activity and promoted healthy eating. Levels of intervention ranged from individual-focused strategies (e.g., one-on-one or telephone counselling) to group sessions or classes. Interventions also varied by their modality, which refers to the method used to deliver the intervention; this could be educational, behavioural, or environmental. The duration and intensity of an intervention are also defining characteristics. They ranged from 8 weeks to 24 months with weekly or biweekly interventions, one 5-year-long program, and one lifetime program. The majority of interventions required a sustained, long-term commitment to achieve the desired outcomes. Furthermore, interventions were mainly targeted to minorities (African American and Latino) who are generally the populations most affected by obesity. Thus, some interventions were tailored to their specific needs and cultural context, using “promotoras” or African American churches to deliver the intervention. Most interventions were at the evaluation stage (*n* = 14), three were at the feasibility stage and six at the implementation stage. In the grey literature, funding was typically sourced from the public health sector or donations. Whereas peer-reviewed literature was funded by a variety of government agencies and private foundations, with a significant number of them receiving support from the National Institutes of Health (NIH) and its various sub-agencies.

### Actors and their functions

**Fig. 7:**
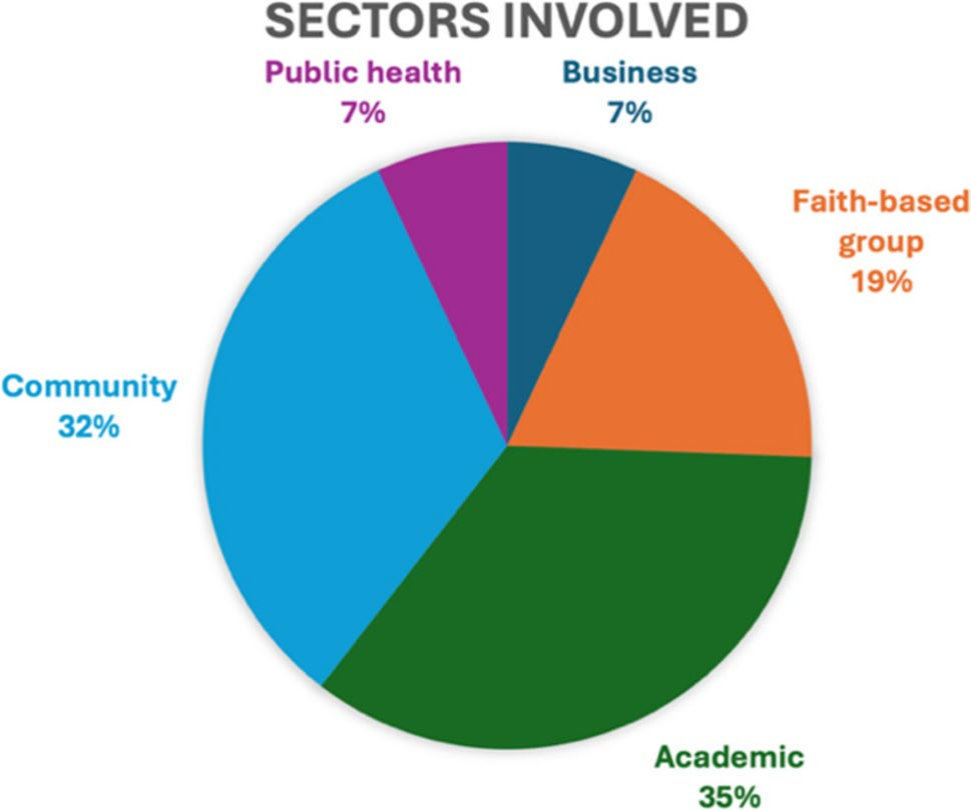
Distribution of sectors involved

The health sector (primary care) has collaborated with various sectors to provide care and services through intersectoral collaboration: Community (food banks, physical activity institutes, community workers, pharmacies, YMCA, local grocery stores, etc.), academic (universities), Faith-based group (church), public health and business (agriculture, technology, disease management company, etc.). Actors from each sector played different roles in interventions, as detailed in Supplemental Material 5. Sample recruitment for the studies was primarily conducted through primary care providers and other community services, including churches. The health sector was represented by physicians, nurses, registered dietitians, psychologists, clinical exercise specialists, and health educators who participated in creating weight management treatment plans for the intervention, monitoring parameters, assessing potential participants, and communicating with them. As shown in Fig. 7, the most significant partnerships are with the academic and community sectors. The academic sector formed the research team, which played key roles in providing technical and substantive expertise and monitoring of interventions. The community remained the most frequent location for intervention delivery; however, a diverse range of locations was also observed. Partnerships were created to offer access to services such as the wellness center, fitness center, nutrition and behavioural therapy classes. The business sector was also used to expand locally grown products and offer better food access. In some interventions, churches served as the lead convening agency in organizing and hosting, as well as delivering education through sermons.

The presence of health advisors from different sectors (community, religious, work, etc.) was a main addition to some selected interventions. The health advisor was a key person who provided education, guided the patient, offered care, and served as a pivot to collaboration. 

**Fig. 8:**
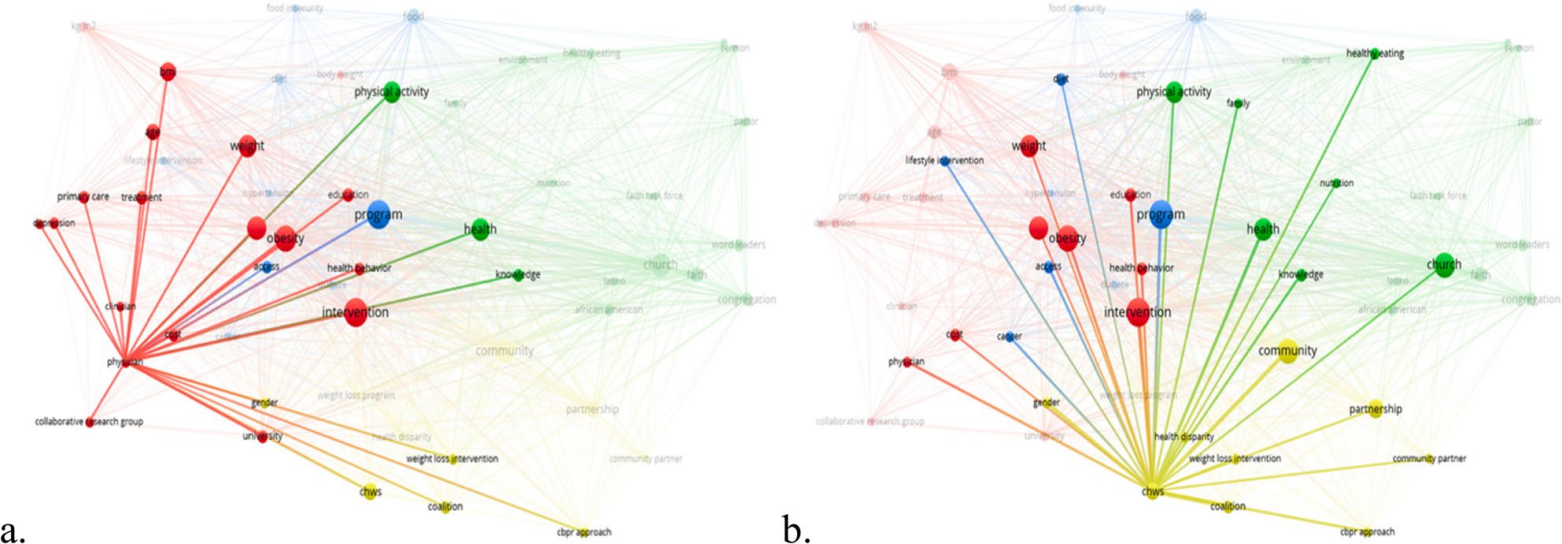
Physician VS community health worker bibliometric links

In Fig. 8.a, the “physician” node (*n* = 28) is the central hub, with strong connections to terms related to the clinical setting, such as “primary care,” “treatment,” “usual care” and specific health conditions. Physicians typically operate within a clinical, disease-focused framework and no link with the “community” node was observed. Supplemental Material 5 shows that their involvement primarily centers on diagnosing obesity, assessing comorbidities, and prescribing medical interventions, such as medications or bariatric surgery. They also provide guidance and education on health behaviours (e.g. diet and physical activity).

In contrast, Fig. 8.b focused on the “CHWs” node (*n* = 94) shows a strong connection to the “community” node (thickness of the link). The total link strength with other nodes for the “CHWs” node is superior to that of the “physician” node, indicating a stronger co-occurrence and greater integration of CHWs in the included literature. CHWs address the social, commercial and environmental determinants of health that physicians may not be equipped to handle [65, 66]. Operating within the community, CHWs serve as a bridge between the clinical setting and broader community support. Supplemental Material 5 shows that they provide culturally sensitive support, help individuals navigate barriers to healthy living, such as food insecurity, and connect them with local resources, including farmers’ markets and exercise programs.

### Outcomes

As shown in Supplemental Material 4, the reported outcome measures were mainly anthropometric measures. They varied widely with no consensus observed, including weight, waist-hip circumference, BMI and body fat percentage. Some studies evaluated metabolic health more extensively through other outcomes such as blood pressure, lipid levels, and HbA1C. In addition, various outcomes were measured in specific interventions, including health outcomes, behavioural outcomes, intervention efficacy, adherence, and satisfaction. Changes in eating behaviours were measured through a food diary app or questionnaires, such as the Quick Food Scan and Fruit, and the Vegetable Scanner developed by the National Cancer Institute, as well as the Eating Inventory and the Brief Questionnaire to Assess Habitual Beverage Intake (BEVQ-15). Physical activity was assessed with questionnaires such as the International Physical Activity Questionnaire (IPAQ) short form, metabolic equivalent task (MET) hours per week, maximal aerobic capacity, activity tracker, and/or sedentary time. Other interventions also assessed mental, emotional, and social well-being through various checklists and scales administered to participants, such as the Utrecht Coping List and the Rosenberg Self-Esteem Scale. In addition, participants were questioned on their perceived health-related quality of life (Weight on Quality of Life-Lite (IWQOL) questionnaire, Patient-Reported Outcomes Measurement Information System-29 (PROMIS-29)) and interviewed to assess their perceptions of program satisfaction and efficacy.

Concerning benefits, most interventions were found to have a modest yet positive effect in decreasing weight over time. This change was significant with longer-term interventions and follow-up.

## Discussion

This scoping review allowed us to identify 23 relevant records that describe intersectoral health interventions for PLO, developed in community and primary care settings. Although no definition of intersectoral collaboration was provided, these interventions are distinguished by a close partnership between actors from diverse sectors and the implication of CHWs. The comparison of scientific and grey literature highlights conceptual and operational divergences, particularly regarding definitions of obesity and collaboration models.

### Redefining the conceptual foundations of obesity

A key observation from this review is the marked heterogeneity in how obesity is defined across sources. The scientific literature remains anchored in the use of body mass index as a primary inclusion criterion, despite growing concerns regarding its limitations in capturing body composition, functional health status, and cardiometabolic risk [67]. In contrast, several grey literature sources challenge the clinical relevance of BMI as a standalone measure and advocate for more nuanced, individualized definitions. These perspectives align with recent frameworks proposed by *The Lancet Commission on Obesity* and Obesity Canada, which differentiate between preclinical obesity (excess adiposity without functional impairment) and clinical obesity (associated with organ or metabolic dysfunction) [11]. They further call for integrating anthropometric measures with assessments of complications, functional status, and quality of life.

This conceptual variability underscores a critical challenge: inadequate or narrow definitions may undermine the appropriateness, acceptability, and effectiveness of interventions [68]. Our findings highlight the need for evaluation frameworks grounded in a life-trajectory approach—models that consider individual health trajectories, social positioning, and the structural DoH [69]. 

### Intersectorality: practice without a clear conceptual anchor

Although intersectoral collaboration was central to the interventions included in this study, none of the documents explicitly defined the concept. Nonetheless, many initiatives reflected varying levels of engagement across sectors, including healthcare, academia, community-based organizations, faith institutions, and businesses, consistent with Himmelman’s continuum of inter-organizational relationships [21], which ranges from networking to close collaboration. Most interventions were situated within the domains of cooperation or collaboration, suggesting a shift away from siloed approaches toward integrated strategies in chronic disease management.

However, we observed differences in how these partnerships were operationalized. Scientific literature often describes more formal, structured collaborations embedded within academic or institutional settings. By contrast, the grey literature highlighted community-led or grassroots initiatives that tended to be less institutionalized and frequently in early stages of implementation. These findings emphasize the importance of clearly articulating intersectoral mechanisms and governance models to support sustainability and replication [70]. 

### Innovations serving contextualized approaches

One of the most salient innovations emerging from the review was the integration of CHWs. Positioned within their communities, CHWs serve as trusted liaisons between clinical services and the social realities of underserved populations. Their roles extend beyond outreach and education; they provide culturally appropriate guidance, address barriers such as stigma and limited access to resources, and promote engagement in health interventions [71, 72]. Unlike physicians, whose roles often remain anchored in biomedical domains, CHWs operate at the intersection of health and social care, facilitating continuity, empowerment, and trust.

Recognizing and institutionalizing the role of CHWs within intersectoral strategies holds promise for advancing more equitable, person-centred models of care. It also highlights the need for adequate training, stable funding, and formalized support structures to ensure their long-term impact [73]. 

### Evaluation challenges: the case for context-sensitive models

Our review identified significant variability in outcome indicators across studies. While most interventions focused on clinical endpoints, such as weight, BMI, and metabolic markers, some also incorporated psychosocial and quality-of-life measures [74]. However, few studies systematically evaluated changes in DoH, despite widespread recognition of their role in shaping obesity risk and outcomes.

This gap represents a critical limitation. Determinants such as housing conditions, food insecurity, income level, and social stigma are not merely contextual variables but central components of the lived experience of obesity [75]. The complex interplay of these DoH illustrates that obesity is not a consequence of individual pathology but is a systemic issue rooted in structural inequalities and the social, commercial and physical environments that shape health outcomes. Embedding these factors as core outcomes, rather than peripheral considerations, would enhance both the explanatory power and the social relevance of intervention evaluations [76]. Our understanding is that a persistent challenge remains in integrating DoH into the medical assessments of PLO by healthcare professionals. In current practice, clinical care often results more in the sharing of information with the social and environmental sectors than in the genuine co-construction of new health practices with actors from different areas of society.

### Recommendations for implementation and research

Considering our findings, several recommendations emerge to guide both implementation and future research. First, there is a pressing need to redefine inclusion criteria and outcome measures. These should extend beyond anthropometric indicators to incorporate functional, psychosocial, and qualitative dimensions, thereby enabling a more comprehensive and nuanced understanding of obesity [11, 77]. Equally important is the clarification and formalization of intersectoral models. Establishing clear definitions, collaborative frameworks, and governance mechanisms will enhance transparency, support scalability, and open pathways to more sustainable funding opportunities [21, 70]. Another priority concerns the institutionalization of CHWs as integral members of multidisciplinary teams. This integration requires stable financing, standardized training, and appropriate oversight to ensure that CHWs are both supported and empowered in their roles [71–73]. Furthermore, the evaluation of interventions should be consistently approached through a SDH lens. By treating structural factors as primary outcomes, it becomes possible to better assess progress toward health equity rather than limiting assessments to individual-level changes [75, 76]. Finally, the adoption of participatory and co-designed approaches is essential. Centring the expertise and priorities of PLO and community partners ensures greater relevance, legitimacy, and sustainability of interventions [69]. Initiatives such as COLLAB-INTER-360-Obesity exemplify the potential of collaborative models that integrate diverse forms of expertise while advancing equity-oriented innovation.

### Strengths and limitations

This is the first scoping review on this topic, providing a unique lens on the design, implementation, and evaluation of intersectoral interventions and community management of obesity, with qualitative findings corroborated by a bibliometric analysis. The review employed a comprehensive search strategy across three peer-reviewed databases and grey literature to identify intersectoral interventions specifically for PLO. To ensure that all relevant studies were included, we employed a broad search strategy and established clear inclusion criteria. However, this review has certain limitations, notably the exclusion of records outside high-income countries and those published in languages other than English or French. This may have led to the omission of relevant documents from specific contexts, such as Spanish-speaking countries in Latin America, where community-based interventions could be particularly impactful [78, 79]. Furthermore, because many terms can be used to describe intersectoral collaboration, it is possible that we did not extract all relevant records in the existing literature. However, the inclusion of grey literature has offered a more comprehensive understanding of intersectoral interventions targeting PLO characteristics. Finally, while the use of automated bibliometric analysis to support qualitative findings follows the innovative trajectory set by Gartner et al. (2022), its fixed data-structuring and visualization parameters represent a methodological constraint. Additional studies are therefore necessary to further explore and validate this hybrid approach [39]. 

## Conclusion

This study provides a comprehensive overview of intersectoral interventions designed for PLO, highlighting the diversity of implementation modalities and evaluation strategies currently in use. Despite growing recognition of obesity as a complex and chronic condition, many interventions remain anchored in biomedical paradigms, with limited responsiveness to the social, cultural, commercial and structural determinants that shape health outcomes. Our findings suggest that truly effective and equitable obesity interventions must extend beyond traditional healthcare settings. They require sustained collaboration across sectors, meaningful engagement with affected populations, and the integration of community health workers as key facilitators of context-sensitive care. Moreover, evaluation models should evolve to include DoH not only as background variables but as primary indicators of success. To accelerate the development of person-centred and equity-oriented approaches, we call for a shift toward clearly defined, structurally supported, and embedded intersectoral frameworks within broader health and social systems. Future efforts should prioritize co-design with communities and align evaluation metrics with real-world outcomes to contribute to more sustainable and appropriate responses to the obesity epidemic.

The results of this study will inform the participants of the COLLAB-INTER-360-Obesity learning community, enabling them to optimize the next stage of this citizen-based participatory action research project. Specifically, they will guide the implementation of co-design workshops for the intersectoral learning community, promoting an integrated and intersectoral perspective on obesity. This approach remains underdeveloped and insufficiently embedded within the healthcare system.

## Supplementary Information

Supplementary Material 1

Supplementary Material 2

Supplementary Material 3

Supplementary Material 4

Supplementary Material 5

Supplementary Material 6

## Data Availability

No datasets were generated or analysed during the current study.

## Abbreviation

PLO: People Living with Obesity
SDoH: Social Determinants of Health
DoH: Determinants of Health
CDoH: Commercial Determinants of Health
PRISMA: Preferred Reporting Items for Systematic reviews and Meta-Analyses
