# Supplementary material for: Intersectoral interventions for people living with obesity: a scoping review and bibliometric analysis
Source: BMC Public Health. 2026 May 30;26:2249. doi: 10.1186/s12889-026-27953-6 (PMC13425862; doi:10.1186/s12889-026-27953-6)
Supplement: Supplementary file 1 — Supplementary Material 1 [file 12889_2026_27953_MOESM1_ESM.docx]

All terms included in the bibliometric map are presented in the table below.

| Cluster 1 - Red | Cluster 2 - Green | Cluster 3 - Yellow | Cluster 4 - Blue |
| --- | --- | --- | --- |
| Age  Bmi  Body weight  Clinician  Collaborative research group  Cost  Depression  Education  Health behavior  Intervention  Kg m^2^  Obesity  Physician  Primary care  Treatment  University  Usual care  Weight  Weight loss | African American  Church  Clergy  Congregation  Environment  Faith  Faith task force  Family  Health  Healthy eating  Knowledge  Latino  Nutrition  Pastor  Physical activity  Sermon  Social support  WORD leaders | CBPR approach  CHWs  Coalition  Community  Community partner  Gender  Health disparity  Partnership  Weight loss intervention  Weight loss program | Access  Cancer  Diabetes  Diet  Food  Food insecurity  Hypertension  Lifestyle intervention  Program |
