# Supplementary material for: Intersectoral interventions for people living with obesity: a scoping review and bibliometric analysis
Source: BMC Public Health. 2026 May 30;26:2249. doi: 10.1186/s12889-026-27953-6 (PMC13425862; doi:10.1186/s12889-026-27953-6)
Supplement: Supplementary file 2 — Supplementary Material 2 [file 12889_2026_27953_MOESM2_ESM.docx]

**Search strategies**

**Medline (Ovid)**

**Search date:** 04/15/2024

**Database limits:** no limits

| **#** | **Search strategy** | **Results** |
| --- | --- | --- |
| 1 | Intersectoral collaboration/ OR Interprofessional relations/ OR Interdisciplinary communication/ OR Interdepartmental relations/ OR Interinstitutional relations/ OR Cooperative Behavior/ | 116145 |
| 2 | (intersector* or inter-sector* or multisector* or multi-sector* or cross-sector* or "Health in all policies" or hiap).ti,kf,kw. or (intersector* or inter-sector* or multisector* or multi-sector* or cross-sector* or "Health in all policies" or hiap).ab. /freq=2 | 3360 |
| 3 | (interprofession* or inter profession* or interdisciplinar* or inter disciplinar* or interdepartment* or inter department* or interinstitution* or inter institution* or interagenc* or inter-agenc* or multidisciplinar* or multi disciplinar* or cross-disciplinar*).ti,kf,kw. or (interprofession* or inter profession* or interdisciplinar* or inter disciplinar* or interdepartment* or inter department* or interinstitution* or inter institution* or interagenc* or inter-agenc* or multidisciplinar* or multi disciplinar* or cross-disciplinar*).ab. /freq=2 | 66622 |
| 4 | ((collaborati* or cooperati* or holistic or coordinat* or integrat* or comprehensive* or collectiv*) adj2 (approach* or intervention* or engag* or relation* or communicat*)).ti,kf,kw. or ((collaborati* or cooperati* or holistic or coordinat* or integrat* or comprehensive* or collectiv*) adj2 (approach* or intervention* or engag* or relation* or communicat*)).ab. /freq=2 | 17663 |
| 5 | (partnering or partnership* or coalition* or alliance*).ti,kf,kw. or (partnering or partnership* or coalition* or alliance*).ab. /freq=2 | 33037 |
| 6 | Delivery of Health Care, Integrated/ | 14462 |
| 7 | (integrated adj3 (care or healthcare or delivery or framework*)).tw,kf,kw. | 22886 |
| 8 | (clinical adj3 community).tw,kf,kw. | 6594 |
| 9 | Chronic Care Model.tw,kf,kw. | 1041 |
| 10 | or/1-9 | 250225 |
| 11 | *obesity/ or *obesity hypoventilation syndrome/ or *obesity, abdominal/ or *obesity, maternal/ or *obesity, metabolically benign/ or *obesity, morbid/ | 166983 |
| 12 | obesity.ti,kf,kw. or obesity.ab. /freq=3 | 179147 |
| 13 | (obese adj2 (patient* or population or adult* or men or women or male* or female*)).ti,kf,kw. | 18529 |
| 14 | 11 OR 12 OR 13 | 252539 |
| 15 | 10 AND 14 | 2142 |
| 16 | (newborn* or new-born* or neonat* or neo-nat* or infan* or child or children* or childhood* or adolesc* or paediatr* or pediatr* or baby* or babies* or toddler* or kid or kids or boy* or girl* or juvenile* or teen* or youth* or pubescen* or preadolesc* or prepubesc* or preteen or tween).ti,kf,jw. not (adult* or elder or elders or elderly* or middle-age*).ti,kf. | 2180992 |
| 17 | 15 not 16 | 1350 |
| 18 | limit 17 to yr=2006-2024 | 1250 |

**CINAHL (EBSCO)**

**Search date:** 04/15/2024

**Database limits:** no limits

| **#** | **Search strategy** | **Results** |
| --- | --- | --- |
| 1 | (MH "Collaboration") OR (MH "Interprofessional Relations") OR (MH "Interdepartmental Relations") OR (MH "Interinstitutional Relations") OR (MH "Community-Institutional Relations") OR (MH "Cooperative Behavior") | 100,605 |
| 2 | TI (intersector* or inter-sector* or multisector* or multi-sector* or cross-sector* or "Health in all policies" or hiap) | 1,004 |
| 3 | TI (interprofession* or inter profession* or interdisciplinar* or inter disciplinar* or interdepartment* or inter department* or interinstitution* or inter institution* or interagenc* or inter-agenc* or multidisciplinar* or multi disciplinar* or cross-disciplinar*) | 24,416 |
| 4 | TI ((collaborati* or cooperati* or holistic or coordinat* or integrat* or comprehensive* or collectiv*) N2 (approach* or intervention* or engag* or relation* or communicat*)) | 7,192 |
| 5 | TI (partnering or partnership* or coalition* or alliance*) | 19,667 |
| 6 | (MH "Health Care Delivery, Integrated") | 15,527 |
| 7 | TI (integrated N3 (care or healthcare or delivery or framework*)) | 6,296 |
| 8 | TI (clinical N3 community) | 998 |
| 9 | TI Chronic Care Model | 480 |
| 10 | S1 OR S2 OR S3 OR S4 OR S5 OR S6 OR S7 OR S8 OR S9 | 154,768 |
| 11 | (MM "Obesity") OR (MM "Obesity, Maternal") OR (MM "Obesity, Morbid") | 64,050 |
| 12 | TI obesity | 44,693 |
| 13 | TI (obese N2 (patient* or population or adult* or men or women or male* or female*)) | 8,636 |
| 14 | S11 OR S12 OR S13 | 84,050 |
| 15 | S10 AND S14 | 848 |
| 16 | TI (newborn* or new-born* or neonat* or neo-nat* or infan* or child* or adolesc* or paediatr* or pediatr* or baby* or babies* or toddler* or kid or kids or boy* or girl* or juvenile* or teen* or youth* or pubescen* or preadolesc* or prepubesc* or preteen or tween) OR SO (pediatr* or paediatr*) NOT TI (adult* OR elder OR elders OR elderly* OR middle-age*) | 810,822 |
| 17 | S15 NOT S16 | 523 |
| 18 | S17 AND PY 2006-2024 | 488 |

**Web of Science (EBSCO)**

**Search date:** 04/15/2024

**Database limits:** no limits

| **#** | **Search strategy** | **Results** |
| --- | --- | --- |
| 1 | TI=(intersector* OR "inter-sector*" OR multisector* OR "multi-sector*" OR "cross-sector*" OR "Health in all policies" OR hiap) | 4,576 |
| 2 | TI=(interprofession* OR "inter profession*" OR interdisciplinar* OR "inter disciplinar*" OR interdepartment* OR "inter department*" OR interinstitution* OR "inter institution*" OR interagenc* OR "inter-agenc*" OR multidisciplinar* OR "multi disciplinar*" OR "cross-disciplinar*") | 81,707 |
| 3 | TI=((collaborati* OR cooperati* OR holistic OR coordinat* OR integrat* OR comprehensive* OR collectiv*) NEAR/2 (approach* OR intervention* OR engag* OR relation* OR communicat*)) | 50,964 |
| 4 | TI=(partnering OR partnership* OR coalition* OR alliance*) | 130,823 |
| 5 | TI=(integrated NEAR/3 (care OR healthcare OR delivery OR framework*)) | 16,444 |
| 6 | TI=(clinical NEAR/3 community) | 1,974 |
| 7 | TI="Chronic Care Model" | 294 |
| 8 | #1 OR #2 OR #3 OR #4 OR #5 OR #6 OR #7 | 283,689 |
| 9 | TI=obesity | 141,815 |
| 10 | TI=(obese NEAR/2 (patient* OR population OR adult* OR men OR women OR male* OR female*)) | 30,023 |
| 11 | #9 OR #10 | 171,224 |
| 12 | #8 AND #11 | 870 |
| 13 | TI=(newborn* or new-born* or neonat* or neo-nat* or infan* or child or children* or childhood* or adolesc* or paediatr* or pediatr* or baby* or babies* or toddler* or kid or kids or boy* or girl* or juvenile* or teen* or youth* or pubescen* or preadolesc* or prepubesc* or preteen or tween) NOT TI=(adult* or elder or elders or elderly* or middle-age*) | 2,319,357 |
| 14 | #12 NOT #13 | 541 |
| 15 | #14 AND PY=2006-2024 | 479 |
