# Supplementary material for: Intersectoral interventions for people living with obesity: a scoping review and bibliometric analysis
Source: BMC Public Health. 2026 May 30;26:2249. doi: 10.1186/s12889-026-27953-6 (PMC13425862; doi:10.1186/s12889-026-27953-6)

Search for a conceptual framework regarding the factors that influence an individual's overall health

Ovid MEDLINE(R) ALL <1946 to August 07, 2024>
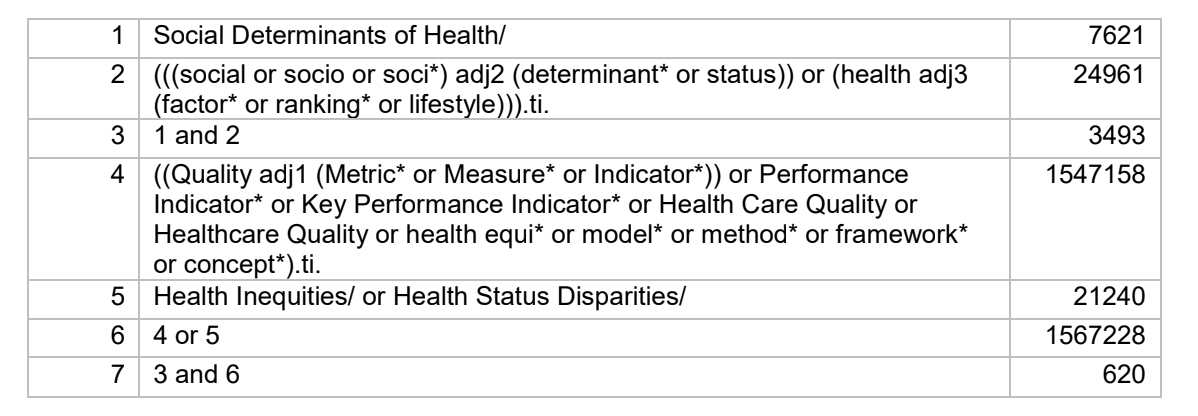

Supplement: Supplementary file 6 — Supplementary Material 6 [file 12889_2026_27953_MOESM6_ESM.docx]
